# Supplementary material for: Misjudgement of One’s Own Performance? Exploring Attention Deficit (Hyperactivity) Disorder (ADHD) and Individual Difference in Complex Music and Foreign Language Perception
Source: Int J Environ Res Public Health. 2023 Sep 27;20(19):6841. doi: 10.3390/ijerph20196841 (PMC10572614; doi:10.3390/ijerph20196841)
Supplement: Supplementary file 1 [file ijerph-20-06841-s001.zip › ijerph-2560520-supplementary.pdf]

International Journal of
Environmental Research and Public Health

MDPI

Table S1. illustrates the correlations of the perceptual language and musical variables for all groups.

	Self-estimation musical ability	Degree of certainty
**Perceptual language ability**	−0.04	0.13
**Perceptual musical ability**	0.32 **	0.19

** $p < 0.001$ (uncorrected, two-tailed).

Table S2. shows the correlations of the perceptual language and musical variables for the musically naïve and the music-educated groups.

	Self-estimation musical ability	Degree of certainty
**Perceptual language ability**	0.17	0.37 **
**Perceptual musical ability**	0.65 **	0.05

** $p < 0.001$ (uncorrected, two-tailed).

Table S3. shows the correlations of the outcome variables and the discriminant functions of the variables of the investigation. We used an arbitrary cutoff of 0.40 to decide which of the variables were large enough to discriminate the groups.

	Function 1	Function 2
**Perceptual musical ability**	0.67 *	0.43
**Short term memory capacity**	0.43 *	0.23
**Perceptual language ability**	0.40 *	0.27
**Self-estimation musical ability**	−0.08	0.79 *
**Degree of certainty**	0.18	−0.56 *

* Largest absolute correlation between each variable and any discriminant function.
